# Protective Role of Ice Barriers: How Reproductive Organs of Early Flowering and Mountain Plants Escape Frost Injuries

**DOI:** 10.3390/plants10051031

**Published:** 2021-05-20

**Authors:** Clara Bertel, Jürgen Hacker, Gilbert Neuner

**Affiliations:** 1 Department of Botany, University of Innsbruck, Sternwartestrasse 15, 6020 Innsbruck, Austria; gilbert.neuner@uibk.ac.at; 2 Hechenbichler GmbH, Cusanusweg 7-9, 6020 Innsbruck, Austria; j.hacker@amalgerol.com

**Keywords:** freezing stress, ice nucleation, ice propagation, infrared differential thermal analysis, reproductive success

## Abstract

In the temperate zone of Europe, plants flowering in early spring or at high elevation risk that their reproductive organs are harmed by episodic frosts. Focusing on flowers of two mountain and three early-flowering colline to montane distributed species, vulnerability to ice formation and ice management strategies using infrared video thermography were investigated. Three species had ice susceptible flowers and structural ice barriers, between the vegetative and reproductive organs, that prevent ice entrance from the frozen stems. Structural ice barriers as found in *Anemona nemorosa* and *Muscari* sp. have not yet been described for herbaceous species that of *Jasminum nudiflorum* corroborates findings for woody species. Flowers of *Galanthus nivalis* and *Scilla forbesii* were ice tolerant. For all herbs, it became clear that the soil acts as a thermal insulator for frost susceptible below ground organs and as a thermal barrier against the spread of ice between individual flowers and leaves. Both ice barrier types presumably promote that the reproductive organs can remain supercooled, and can at least for a certain time-period escape from effects of ice formation. Both effects of ice barriers appear significant in the habitat of the tested species, where episodic freezing events potentially curtail the reproductive success.

## 1. Introduction

As reproductive organs of plants are most sensitive to freezing [1], and especially the style and ovules [2], flowering is usually restricted to frost free-periods. However, some species cannot completely escape freezing temperatures during flowering. In the temperate zones of Europe, mountain and early season flowering plants can be hit by episodic frosts throughout the whole reproductive development [1,3,4]. Depending on the particular habitat, such episodic frosts can occur regularly: In the alpine life zone, temperature minima of air during the snow free period are between −1.0 and approximately −20 °C, as reviewed in [4]. In the inn valley, night frosts are possible until the middle of May and from September onwards [4]. Due to climate change spring temperatures are becoming warmer and snowmelt is occurring earlier in spring, leading to an advanced development in many plant species and thereby increasing risk that plants are harmed by episodic frost events during their reproductive development [5,6,7]. To avoid the risk of damage by ice formation in reproductive tissues, different functional strategies that finally allow securing reproductive success have evolved [6].

While little is known about ice susceptibility of reproductive organs of early flowering plants [1], most mountain plants were shown to be ice sensitive [3]. As the only exception, reproductive organs of the nival species *Ranunculus glacialis* L. could be proven to tolerate ice formation without freezing injury [3]. Frost survival of reproductive organs in all other alpine species investigated until now relied on avoidance of ice formation, i.e., a strategy termed supercooling. Vegetative plant parts usually show little supercooling capacity in nature and ice forms at mild subzero temperatures [8]. Ice spreading occurs fast, at rates of up to 27 cm·s^−1^ into all plant parts that are colder than 0 °C [9,10]. The challenge is to prevent the spread of ice into the ice sensitive reproductive organs, and, as well, to inhibit intrinsic ice nucleation in the supercooled reproductive organs. For reproductive organs, two ice barrier types have been described that stop ice penetration either structurally or thermally. Protective structural (anatomical) ice barriers between the stem and the flowers that prevent ice propagation from the stem into the freezing sensitive flower organs were first found in woody fruit crops [11,12]. In alpine woody dwarf shrubs, such structural ice barriers persist during shoot elongation, anthesis, and fruiting throughout summer [13,14]. The naturally occurring temperature gradient within high alpine cushions interrupted ice propagation thermally (*Saxifraga bryoides* L., *Saxifraga caesia* L., *Saxifraga moschata* Wulfen and *Silene acaulis* (L.) Jacq.; [15]) and every reproductive shoot on the cushion needed an autonomous ice nucleation event to initiate freezing, which facilitates frost survival by supercooling. Similarly, burial of the interconnecting shoot several centimeters below the soil surface creates a thermal barrier against ice spread between single leaves and flowers [8]. Generally, little is known how herbaceous early-flowering and mountain species manage ice spread around and into reproductive organs. Similarly, it is unclear whether and how much reproductive organs of early flowering plants are damaged after the formation of ice.

We hypothesized that species whose reproductive organs and processes are threatened by episodic freezing events should either show structural or thermal ice management to prevent ice formation in reproductive organs. Infrared video thermography has proven to be a particularly suitable method for investigating ice management strategies at the organ and whole plant level [9,14,15]. Infrared video thermography does not necessarily allow conclusions to be drawn about the exact location of the ice nucleation in the tissue, but the spread of the ice, ice barriers, and supercooling of organs or tissues can be nicely depicted. Investigations were supplemented by a controlled frost test, evaluating the sensitivity of flowers to ice-formation, and anatomical investigations, visualizing the anatomical structure in the area of putative ice barriers. In the present study, the specific ice management strategies were investigated in whole plants, vegetative and reproductive organs of two early-flowering mountain plants (*Anemone nemorosa* L., *Jasminum nudiflorum* Lindl.) and three herbaceous early spring bloomers (*Galanthus nivalis* L., *Muscari* sp., *Scilla forbesii* (Baker) Speta), each representing a specific life or growth form.

## 2. Results

### 2.1. Ice Management Strategies

Ice management strategy, susceptibility to freezing, and ice nucleation temperatures differed across the studied species and organs (Table 1). While leaves and shoots were ice tolerant in all species, flowers were ice tolerant in only two of the investigated species, i.e., *G. nivalis* and *S. forbesii*. Structural ice barriers between the stem and flowers were present in three species whose flowers were ice susceptible. Ice nucleation temperatures varied among the organs of one species (further details in Section 2.2) and the different species (Supplementary Table S1). The soil acted as a thermal insulator in all species. The whole plant freezing patterns were highly species-specific and are described in detail in the following sections.

### 2.2. Species-Specific Freezing Patterns

#### 2.2.1. *Anemone nemorosa* L.

**Figure 1:**
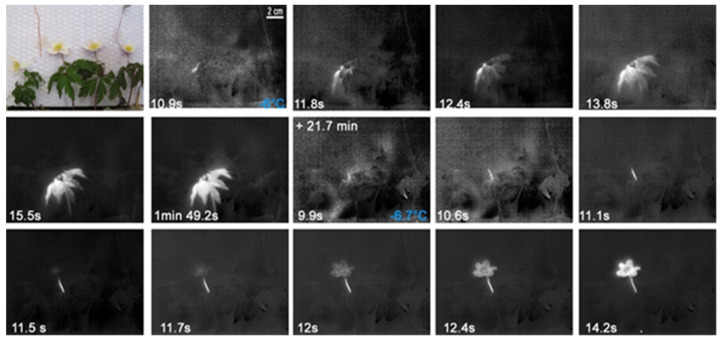
Digital image and chronological sequence of IDTA images obtained on four *Anemone nemorosa* L. individuals. The first ice nucleation occurred in a blade of a bract at −6 °C. From there ice spread into all bracts and into the stem beneath. Under the experimental conditions, the flower only froze some time later (+21.7 min) after a second ice nucleation event in the pedicel at −6.7 °C. On the bottom left corner, the time since the respective ice nucleation event is shown.

The freezing pattern of *A. nemorosa* individuals suggests a structural ice barrier in the node of the flowering stem where three petiolate leaf-like bracts are inserted in a whorl (Figure 1). Under the experimental conditions, the first ice nucleation occurred in one of the four specimens in a leaf blade at −4.9 °C. From there, the ice spread into the other leaf blades and into the stem beneath. Ice nucleation in the leaves of the other individuals occurred at slightly lower temperatures (Supplementary Table S1), as well as in the leaf blade or in a petiole. In all specimens, the flower and the pedicel only froze upon a second ice nucleation event later and at lower temperatures than the bracts and the stem beneath. This second ice nucleation event was initiated in one case in the flower and in all others in the pedicel, as shown in Figure 1. Overall, ice nucleation in bracts occurred at higher temperatures than in flowers (F_1,3_ = 36.98, *p* = 0.009). Once nucleated, the flowers were frost killed, as they do not have ice tolerance (see Table 1).

#### 2.2.2. *Galanthus nivalis* L.

**Figure 2:**
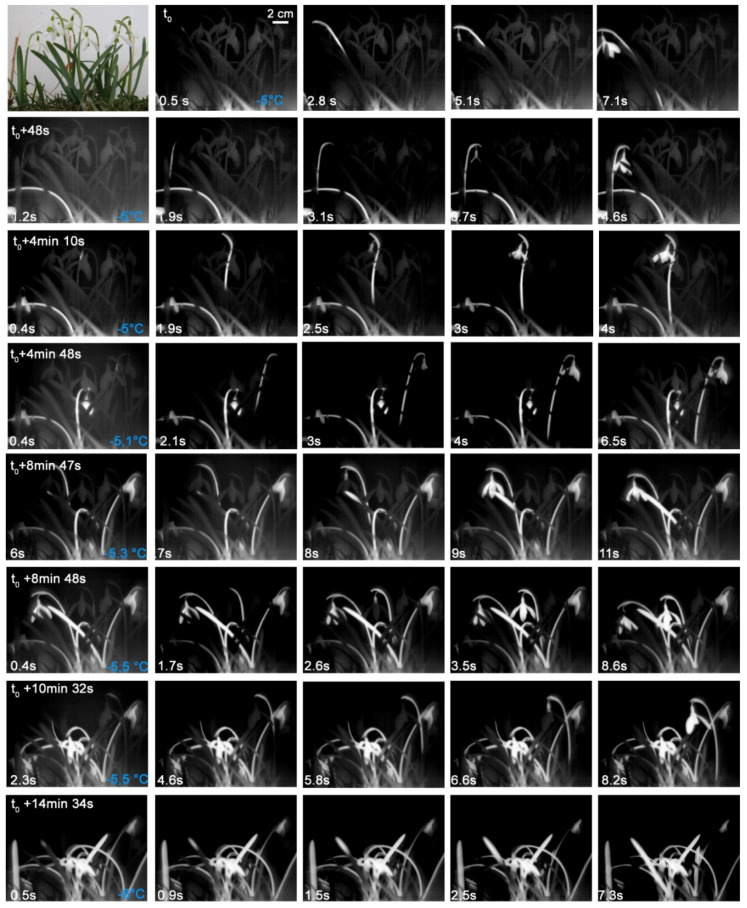
Digital image and sequence of infrared images recorded on seven *Galanthus nivalis* L. individuals that were exposed to a controlled freezing treatment within an excavated lawn piece. Flowers shoots and leaves froze separately from each other. The ice nucleation in the flowering shoots occurred between −5.0 and −5.5 °C and mostly took place in the bract or in the pedicel. The leaves froze at slightly lower freezing temperatures. The ice nucleation mostly occurred in the upper part of the leaf blade. Ice nucleation temperatures are given in blue in the bottom right corner of the first column. On the bottom left corner, the time since the respective ice nucleation event is shown.

**Figure 3:**
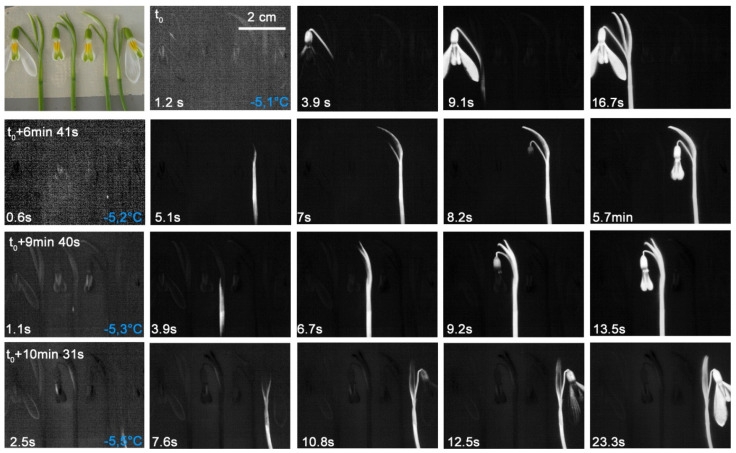
Digital image and infrared image sequence measured on four detached flowering shoots of *Galanthus nivalis* L. during a controlled frost treatment. The ice nucleation took place in the pedicel of the first plant and in the flower stem of all others and took place between −5.1 and −5.5 °C. The ice nucleation mostly occurred in the upper part of the bract. Ice nucleation temperatures are given in blue in the bottom right corner of the first column. On the bottom left corner, the time since the respective ice nucleation event is shown.

In *G. nivalis*, ice propagation patterns were determined under different experimental settings. In the first experiment, the spread of the ice was measured in seven intact *G. nivalis* plants that had been dug out as a whole within a lawn piece. This freezing treatment comes close to natural night frosts where the above ground plant parts freeze while temperatures in the soil remain above 0 °C. As the infrared images show, the flowering shoot and the leaves froze separately from each other (Figure 2). The flowering shoot usually froze at higher freezing temperatures than the leaves (F_1,26_ = 22.14, *p* < 0.001). Ice nucleation mostly took place in the bract-like spathe or in the pedicel and occurred in a narrow temperature range between −5.0 °C and −5.7 °C (Figure 2 and Figure 3). The ice nucleation in the leaves usually took place near the tip of the leaf or in the upper third, as the temperature was higher near the ground. The leaves froze between −4.8 and −6.8 °C. Despite exposure to a freezing temperature down to −8.9 °C, the plants readily survived and flowers have shown to be ice tolerant (Table 1).

In a second experiment, the spread of ice was measured in four detached flowering shoots of *G. nivalis*. As the infrared images show (Figure 3), ice nucleation occurred in one case in the pedicel and in all others in the flower stem. The ice spread continuously into pedicel, the flower and the tepals. The freezing occurred in a similar, narrow temperature range between −5.1 and −5.5 °C as in the previous experiment with whole plants inside the lawn piece (see Figure 2).

**Figure 4:**
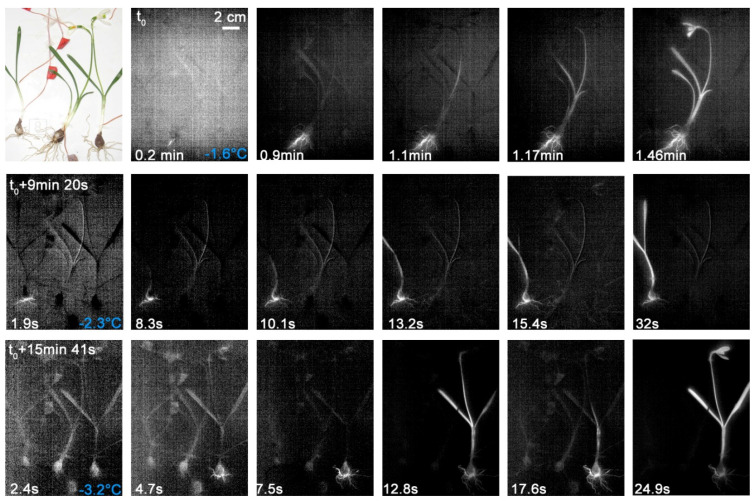
Digital image and infrared image sequence measured on three excavated individuals of *Galanthus nivalis* L. during a controlled frost treatment. The ice nucleation always took place in the area of the roots. The ice spread throughout the plant without an ice barrier. Ice nucleation temperatures are given in blue in the bottom right corner of the first column. On the bottom left corner, the time since the respective ice nucleation event is shown.

In a third experiment, three intact individuals of *G. nivalis* were excavated and the soil was completely removed. As the infrared image sequences show, ice nucleated in the roots in two individuals (Figure 4). In one individual localization or the ice nucleation event could not be resolved exactly, but was either in a root at the bulb base or in the bulb base itself (Figure 4). After ice nucleation in the root, ice spread barrier-free into other roots, the bulb, the leaves, and through the flower stem and pedicel into the flower. Ice nucleation in the roots took place at much higher temperatures, i.e., between −1.6 and −3.2 °C, than in the other two experiments (see Figure 2 and Figure 3). The soil thermally insulates the below-ground organs and the shoot, where all leaves and the flower stem lie close together within a leaf sheath, providing a thermal ice barrier. Ice nucleation temperatures obtained by all three experiments with *G. nivalis* showed that roots, leaves, and flowers froze at significantly different temperatures (F_2,32_ = 97.83, *p* < 0.001) with roots freezing at highest (Est. −2.36 ± 0.28, t = 10.08, *p* < 0.001), flowers at intermediate (Est. −5.23 ± 0.13, t = −39.80, *p* < 0.001), and leaves at lowest temperatures (Est. −6.07 ± 0.16, t = −5.21, *p* < 0.001).

#### 2.2.3. *Jasminum nudiflorum* Lindl.

**Figure 5:**
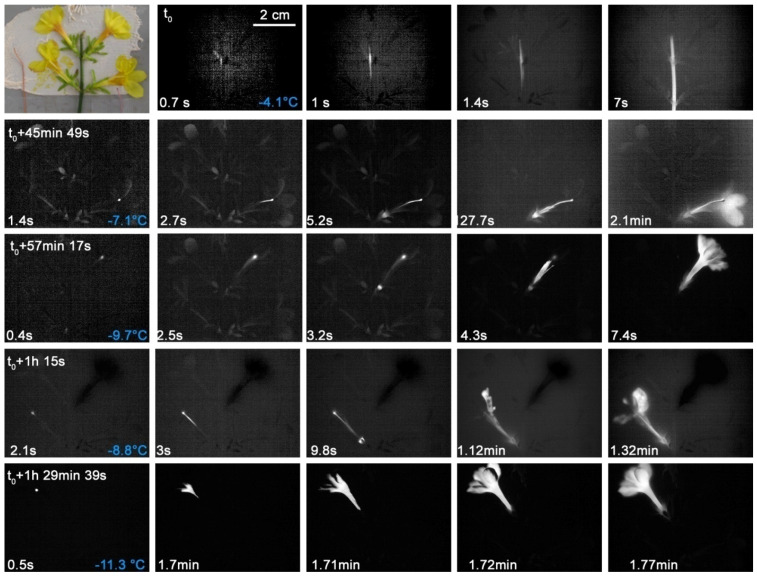
Digital image and IDTA image sequence obtained on a twig of *Jasminum nudiflorum* Lindl. with four flowers monitored during a controlled freezing treatment. The stem froze separately from the flowers. Each flower froze at successively lower freezing temperatures after another separate ice nucleation event (in the top left corner the time span since the initial freezing in the shoot is given.) In three flowers, ice nucleation took place in the stigma. From there, the ice spread over the stylus into the ovary and then into the tepals. Only in the flower that froze latest did the ice nucleation initially take place in a tepal. Ice nucleation temperatures are given in blue in the bottom right corner of the first column. On the bottom left corner, the time since the respective ice nucleation event is shown.

In twigs of *J. nudiflorum* intrinsic structural ice barriers prevented immediate spread of ice from the initially freezing stem into the flowers. The IDTA recordings focus on a twig with four flowers (Figure 5). The stem froze at −4.1 °C but without ice spreading into the flowers. Under the experimental conditions, the flowers did not freeze before −7.1 or latest at −11.3 °C. In three of the four flowers, ice nucleation occurred in the stigma. The ice then spread over the stylus into the ovary and from there into the tepals. Only in one out of four flowers did ice nucleation take place in a tepal. The spread of ice into the inner flower organs could not be depicted in the IDTA images, as the heat released by freezing of the tepals probably masked that coming from the inner flower parts. Supercooled flowers of *J. nudiflorum* survived uninjured but became immediately frost injured when ice nucleated (Table 1).

#### 2.2.4. *Muscari* sp.

**Figure 6:**
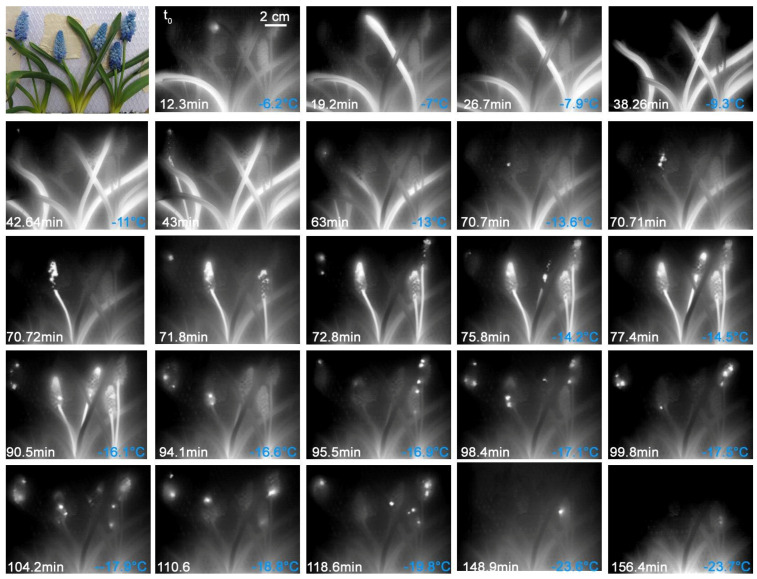
Digital image and infrared image sequence of above-ground parts of potted *Muscari* sp. plants. The leaves freeze at higher temperatures than the inflorescences (The exception is a single flower in the first picture of the sequence). Then the inflorescences with single flowers freeze. Most of the flowers only freeze later after significant supercooling at much lower temperatures. Ice nucleation temperatures are given in blue in the bottom right corner. On the bottom left corner, the elapsed time since the first ice nucleation event is shown.

**Figure 7:**
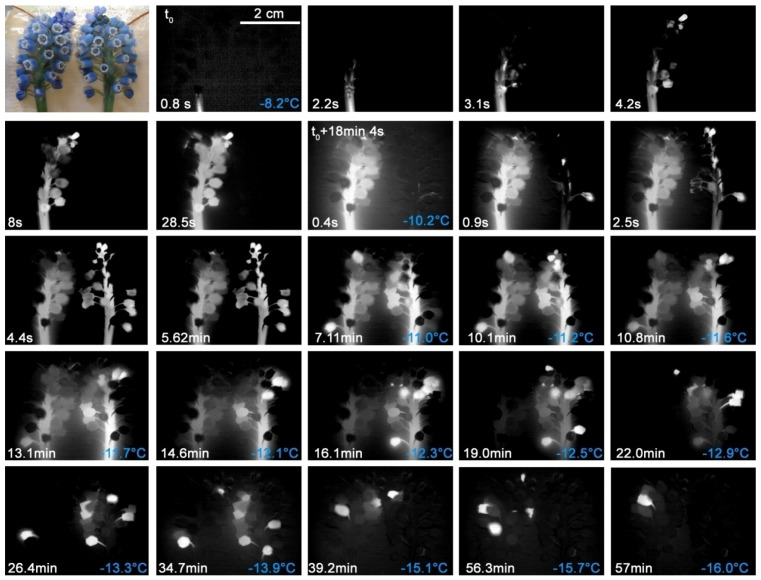
Digital image and sequence of IDTA images during the ice spread in two inflorescences of *Muscari* sp. The ice nucleation took place in the inflorescence axis. From there the ice spread into a few flowers. Only later, at lower temperatures, did the remaining flowers freeze out. Ice nucleation temperatures are given in blue in the bottom right corner. On the bottom left corner, the elapsed time since the first ice nucleation event is shown.

In an experimental approach, similar to the experiment with *G. nivalis,* where lawn pieces were frozen, whole plants of *Muscari* sp. including the soil balls were frost treated. Again, a freezing pattern where leaves and inflorescence froze independently from each other was observed (Figure 6). The inflorescences froze in a wide temperature range between −7.3 and −15.2 °C (see Figure 6 and Figure 7). Ice mostly nucleated in a single flower of the inflorescence, from which the ice then spread into the respective inflorescence axis and in single flowers, often into the young terminal flowers. Only in one case did ice nucleate in the inflorescence axis per se. Strikingly, most of the flowers of an inflorescence did not freeze until much later, at significantly lower freezing temperatures between −7 and −23.6 °C than the main axis of the inflorescence. The freezing temperatures of the flowering stem and that of the flowers was significantly different (F_1,218_ = 101.6, *p* < 0.001). The ice tolerance test revealed that the flowers are ice susceptible (Table 1). Under the experimental conditions, it remains unclear whether the ice barrier breaks or whether a separate ice nucleation event occurred in the respective flower. 

In a second experiment, ice propagation was observed at higher resolution using a macro lens (Figure 7). The leaves froze in a range between −5.3 and −11.1 °C. The ice nucleation in the leaf blade mostly took place in the upper area or near the leaf tip, as the temperature at the leaf base was slightly higher at ground level. In both inflorescences, the ice nucleation took place in the inflorescence axis. From there the ice spread into approximately 11–12 flowers. The ice nucleation temperatures were at −8.2 and −10.2 °C. All other flowers remained supercooled and only froze later, each after individual ice nucleation events in the flower or the pedicel, or a later spread of ice from the frozen inflorescence axis. There are probably efficient ice barriers between the inflorescence axis and most of the flowers. The freezing temperatures at which the supercooled flowers froze were between −9.3 and −17.6 °C.

#### 2.2.5. *Scilla forbesii* (Baker) Speta

**Figure 8:**
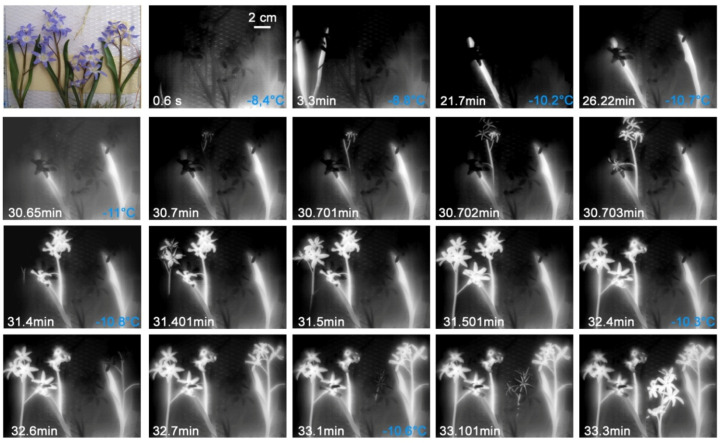
Digital image and sequence of infrared images during ice spreading in four individuals of *Scilla forbesii* (Baker) Speta, which were excavated within a lawn tile and as such subjected to a controlled freezing treatment. Leaves and inflorescences freeze independently. After ice nucleation in a flower, in a pedicel or in the inflorescence stems, the ice spread without interruption into the entire inflorescence. Ice nucleation temperatures are given in blue in the bottom right corner. On the bottom left corner, the elapsed time since the first ice nucleation event is shown.

*S. forbesii* plants that had been excavated within a lawn piece and subjected to a controlled freezing treatment showed again separate freezing of the leaves and the flowers (Figure 8). The unfrozen, warmer soil apparently acts as a thermal ice barrier. The inflorescences froze completely without interruption after a single ice nucleation event. No specific locus of ice nucleation could be detected as it took place in the flower or in the stem of the inflorescence. The ice nucleation temperatures were in the range of −10.3 and −11.0 °C. The ice spread throughout the leaf blades immediately after ice nucleation. The temperature range wherein ice nucleated in the leaves varied largely. The first ice nucleation took place at −8.4 °C and the last was below −11.3 °C. Ice nucleation temperatures of inflorescence stems and leaves did not differ (F_1,7_ = 1.53, *p* = 0.26). The flowers of *S. forbesii* are ice tolerant (Table 1).

### 2.3. Anatomy of Structural Ice Barriers

**Figure 9:**
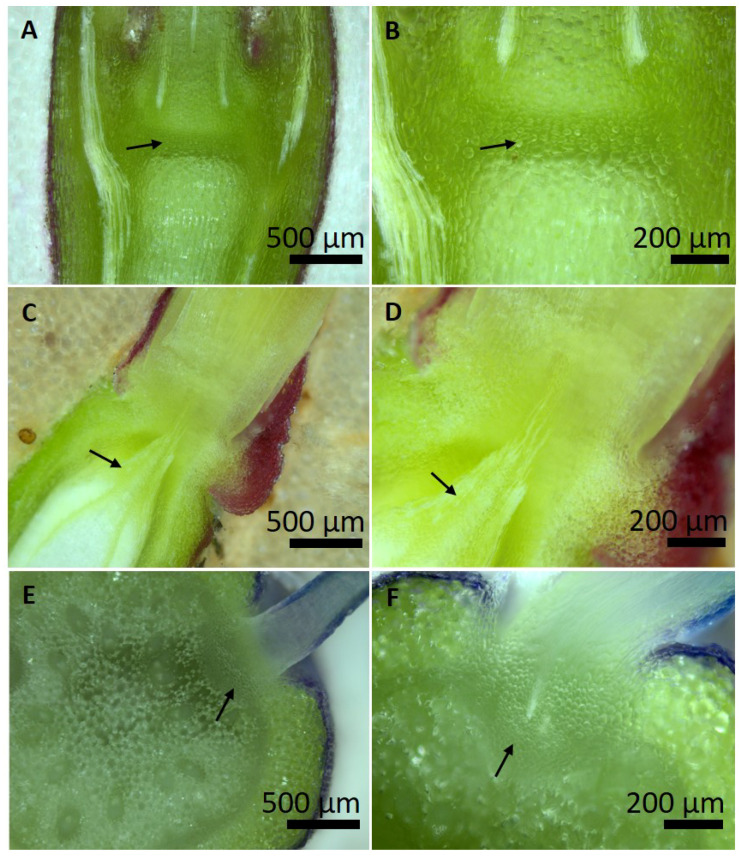
Anatomy of the constriction zone, where ice barriers are located according to IDTA. (**A**,**B**) The region of the nodium of the flowering stem of *Anemone nemorosa* L. (longitudinal section). (**C**,**D**) The connection zone between the stem (lower part) and the pedicel of the flower (upper part) in *J. nudiflorum* Lindl. (longitudinal section). The xylem seems to be narrowed in this zone. (**E**,**F**) Attachment zone of the inflorescence axis and the pedicel of one individual flower in *Muscari* sp. (horizontal section). Arrows point to small tightly packed cells, which likely impede the spread of ice into the flowers.

In the species where a structural ice barrier was found (*A. nemorosa*, *J. nudiflorum* and *Muscari* sp.), the tissue in the respective area was examined microscopically (Figure 9). In all species in this region, a pronounced constriction zone is detectable. The tissues lack intercellular spaces and consist of small, tightly packed cells with thickened cell walls.

## 3. Discussion

The studied species inhabit environments or ecological niches where escape from episodic frost events during the reproductive development is not possible. The measured freezing patterns in flowering individuals reveal the presence of either thermal or structural ice barriers protecting the reproductive organs from an ice entrance. In addition, flowers of two early-flowering species, which lack a structural ice barrier, could tolerate the formation of ice without damage in a similar way to the nival species *R. glacialis* [3].

### 3.1. Structural Ice Barriers

In three of the investigated species, structural ice barriers could be identified by IDTA. Susceptible flowers were only able to survive episodic night frosts below the nucleation point of vegetative tissues by supercooling the ice, as indicated by a clear damage of flowers, seen after icing during controlled freezing down to −5 °C (Table 1). The existence of a structural ice barrier in herbaceous species (*A. nemorosa* and *Muscari* sp.) has not been described before. The observation of an ice barrier between the stem and flowers of *J. nudiflorum* corroborates earlier findings for woody fruit crops [11,12] and alpine dwarf shrubs [13,14].

In *A. nemorosa*, the lower part of the flowering stem and the bracts freeze separately from the pedicel and the flower itself, suggesting an ice barrier at the base of the pedicel. Under the experimental conditions, with a successive lowering of freezing temperatures, the supercooling capacity was evident but did not protect the flowers for a long time (i.e., to temperatures below −8.7 °C, Supplementary Figure S1). At lower, more moderate cooling rates, an even better protection can be expected. In *Muscari* sp. an ice barrier exists at the basis of the pedicel of most flowers, which froze much later at much lower temperatures. In some of these flowers, the separate ice nucleation events in the flower or pedicel appeared to be necessary, in others the ice very likely spread from the inflorescence stem into the flower at lower freezing temperatures. The ice barriers that were obviously present seemed to lose their effectiveness at lower freezing temperatures. In contrast, it was shown that ice barriers observed below the buds of *Picea abies* H. Karst. did not lose their effectiveness at lower freezing temperatures, as ice nucleation of supercooled bud cells was always triggered somewhere inside the bud tissue in distinct distance to the ice barrier [13].

Anatomical features of structural ice barriers have been first described for supercooling reproductive overwintering buds and include small tightly packed cells that are devoid of vacuoles and intercellular spaces, with thick walls containing phenolic compounds (suberin and lignin) or unesterified pectins and regions of dry tissue [16,17,18,19,20,21,22,23,24]. However, in all cases there are only provascular strands [25,26,27,28] and the establishment of xylem continuity during regrowth in spring causes a loss of the ice barrier function. This contrasts to the ice barrier between reproductive and vegetative shoots of *Calluna vulgaris* Hull and alpine dwarf shrubs [13], and also to the investigated species in the present study where a continuous vascular connection into the reproductive shoot is established. In *C. vulgaris*, a 250 μm long constriction zone at the base of the pedicel that lacks pith tissue and intercellular spaces was found and similar structural features as in ice barriers of reproductive buds were present. But in addition, a peculiar anatomical narrowing of the xylem (as also seem to exist in *J. nudiflorum*, Figure 9D) indicated that the diameter of pores in pit membranes of tracheids could be the critical constriction for ice propagation into the persistently supercooled reproductive shoots of *C. vulgaris* [14]. The anatomical nature of structural ice barriers in herbaceous species needs to be further investigated and we did not investigate the peculiar xylem architecture in the region of the ice barriers but a structural constriction zone is clearly visible in all three species (Figure 9). Most structural features of ice barrier tissues reported in earlier studies seem to be present in the investigated species (small size of cells, thick cell walls, lack of huge intercellular spaces, Figure 9), for the xylem we can only speculate but also suggest short vessels with reduced pore diameters of pit membranes. Additionally, a prerequisite for efficient supercooling is the lack of heterogenous ice nucleators or accumulation of anti-ice-nucleating substances [29,30].

### 3.2. Thermal Ice Barriers

The soil acts as a natural thermal ice barrier due to its warmth. While excavated soil-free specimens of *G. nivalis* froze, starting from the root or the root plate, barrier-free throughout without interruption, every leaf and every inflorescence of the *G. nivalis* froze independently from each other when plants were studied in intact lawn pieces. The same pattern was also seen in all other herbaceous species when whole plants in soil were measured (*S. forbesii*, *Muscari* sp., and *A. nemorosa*). This corroborates the recent field findings for a nival species (*R. glacialis*) where soil burial of interconnecting shoots was shown to cause a discontinuous freezing pattern, i.e., each organ froze separately and needed a separate ice nucleation event [8]. Burial of shoots is a characteristic growth feature of high alpine plants which not only ensures frost protection to the shoot and shoot apices itself [31] but at the same time facilitates supercooling of above-ground organs including flowers and can protect from frost damage. In alpine summer and early spring-time, the soil is warmer than air and only freezes during prolonged frosts, since ice in the ground can only spread very slowly [1]. It can be assumed that during short, episodic frosts, the freezing temperatures do not penetrate the soil. The heat storage capacity of the soil was simulated quite well in our night frost experiments as a thermal gradient between the soil that remained frost free and the above ground plant organs as in nature was produced (data not shown). A similar thermal ice barrier has been described for cushion plants, a specific alpine growth form, where the interior of the cushion itself stays above 0 °C during episodic frosts and by this facilitates safe supercooling of inflorescences [15]. This strategy might be particularly important for plants growing on rocks or at sites with hardly any soil.

### 3.3. Ice Nucleation Temperature

Absolute values of ice nucleation temperatures determined in the laboratory are generally weak predictors of the ice nucleation temperature in the field and from this, have only low ecological significance [23]. Under field freezing conditions, most higher plants have ice in their tissue between −0.5 and −3.5 °C ([8], ferns −2.9 to −4.0 °C: [32]), in lab tests, depending on the experimental settings, plant samples mostly supercool to lower freezing temperatures. However, the relative differences between ice nucleation temperatures of reproductive vs. vegetative plant parts provide insights about a higher, equal, or lower supercooling capacity of the particular parts and demonstrate the efficiency of thermal or structural ice barriers. Similarly, differences between species treated by the same experimental procedure allow for comparing their supercooling capacity.

Intraspecific differences in ice nucleation temperatures between vegetative and reproductive organs such as in *J. nudiflorum* where the stem froze at −4.1 °C, and the flowers between −7.1 and −11.3 °C support the protective role of ice barriers (Supplementary Figure S1). In *A. nemorosa* and *Muscari* sp. the pedicels and flowers froze at lower temperatures than the flowering stem, where no difference was found in *S. forbesii* (Supplementary Figure S1). Only in *G. nivalis* did the flowers freeze at higher temperatures than the leaves, in congruence with flowers being ice tolerant (Supplementary Figure S2). These plants, which were excavated within a lawn piece and frost treated as described down to −8.9 °C and buried again outdoors in place were they had been excavated, lacked any visible damage when visited a few days later (Supplementary Figure S2).

Excavated soil-free specimens of *G. nivalis* froze at higher ice nucleation temperatures between −1.6 and −3.2 °C starting from the root or the root plate, while ice nucleated between −4.8 and −6.8 °C in the above ground organs when the roots were in intact soil. In contrast to the epidermis, the rhizodermis of the root represents a poor ice barrier against the entry of ice from outside [33]. If the underground organs were protected from low temperatures by soil, the plant could supercool to distinctly lower freezing temperatures with ice nucleation in the above-ground organs.

Distinctly different ice nucleation temperatures were recorded in the tested species under laboratory conditions (Table 1). While *G. nivalis* froze at comparatively high temperatures between −4.8 and −6.8 °C, the ice nucleation in *A. nemorosa* occurred between −4.9 and −6.6 °C, in *S. forbesii* between −8.4 and −11 °C, and in *Muscari* sp. not before −7.3 and −23.6 °C. Compared by a linear model, only flowers of *A. nemorosa* and flowers of *Muscari* sp., as well as roots of *G. nivalis* differed significantly from the intercept (Table 1, Supplementary Table S1). However, differences in ice nucleation temperatures cannot be interpreted without considering sensitivity to freezing (Table 1).

### 3.4. Freezing Resistance

Supercooling aided by ice barriers can be an important mechanism of frost survival for reproductive organs, even if it is an instable and possibly not a permanent condition. From the infrared recordings, it is not possible to draw any conclusions on the freezing tolerance, as freezing exotherms can originate from harmless extracellular but also lethal intracellular ice formation. The ice tolerance test, however clearly revealed that species with ice barriers have flowers that are ice susceptible. Except for one species, many alpine species were found to be ice susceptible and thus to rely on effective ice barriers in order to survive episodic frosts [3].

In early-flowering plants, frost damage (LT_20_) to leaves has been reported to occur between −4 and −13 °C (*A. nemorosa*, *G. nivalis*: [34]; unpublished Taschler Dunja) and comes close to that reported for temperate alpine species [35,36]. For reproductive organs of early flowering plants, [34] studied flowers of *A. nemorosa* and found that they become frost injured between −6.5 and 8.0 °C, which is close to the frost resistance of flowers of alpine species [3]. Still, as different frost survival mechanisms are involved—supercooling and extracellular ice tolerance—the assessment of freezing resistance in flowers remains a difficult experimental task, as the determination of supercooling capacity requires other experimental settings than when ice tolerance is given. 

## 4. Conclusions

While many plant species of the temperate zone can tolerate the formation of extracellular ice in their generative organs, it is usually lethal in their reproductive organs. Species that flower early in spring seem to have evolved different strategies to deal with the enhanced risk that their reproductive organs are harmed by episodic frost events and to ensure their reproductive success: in some species, whose flowers were sensitive to freezing, a structural ice barrier was observed, which prevents the spread of ice from generative parts into the flowers and thereby allows the flowers to supercool. Other species, which lacked a structural ice barrier, were found to be able to tolerate the formation of extracellular ice in their flowers, which is—to our knowledge—an exception to the majority of the plant species studied. Knowledge about strategies to deal with episodic frosts and their consequences is particularly relevant in the light of recent climate change, which alters snow-pack that despite global warming will increase the probability of exposure to freezing temperatures in late winter and early spring [5,6,7].

## 5. Materials and Methods

### 5.1. Plant Samples

The patterns of ice spread were investigated in five species whose flowering time matches with episodic night frosts. The selected species have differences in growth and life form and in their vertical distribution (Table 2): two mountain plants whose vertical distribution extends into the subalpine life zone, i.e., *A. nemorosa* and *J. nudiflorum* and three colline to montane distributed early spring flowering plants: *G. nivalis*, *Muscari* sp., and *S. forbesii* were studied. All species, except for *Muscari* sp., which was bought as potted plant in a garden market, were sampled in the Botanical Garden in Innsbruck (600 m above sea level, 47°16′14″ N, 11°22′46″ E). All plants were well watered. Species were either excavated with a lawn piece or in case of *J. nudiflorum* twigs were cut off between 16 March 2010 and 26 March 2010 and examined immediately afterwards. Additional investigations were carried out on detached inflorescences of *Muscari* sp. and *G. nivalis*. In the latter, the ice spread was further measured in whole specimens with the soil removed from the bulb and roots. A whole branch with flowers of *J. nudiflorum* was examined.

### 5.2. Freezing Treatment

The samples were treated with freezing temperatures in a chest freezer under controlled temperature conditions. For a recent detailed description of the temperature control system, see [36]. A thermocouple, based on which temperature is measured and controlled, was placed in the middle of the experimental setting, above or just below the investigated flowers. The cooling rate was −5 K·h^−1^ in all experiments, except for the second experiment with *Muscari* sp. In this species, two inflorescences were observed by a makro lens at a cooling rate of −8 K·h^−1^ in order to reduce the long observation time. The freezing treatment started at +2 °C and ended as soon as all parts of the investigated plant were frozen. In addition, further thermocouples were attached to the left and right of the samples at the soil surface, in case the samples were frozen in intact soil and near the flowers. With these thermocouples, absolute sample temperatures were measured, as minor temperature gradients can occur in the exposure chamber of the chest freezer due to positioning of the samples and due heat emitted from the soil. The thermocouples were connected to a data logger (CR10X, Campbell Scientific, Logan, UT, USA), which recorded the temperature data.

### 5.3. Infrared Video Thermography

Ice nucleation and ice spread were measured with a digital infrared camera (FLIR Systems ThermaCAM ™ S60, FLIR Systems AB, Danderyd, Sweden). The use of an infrared camera enables a two-dimensional representation of the heat released by the exothermic process of ice formation. The released heat can be visualized in the infrared image using false colors [9].

The recording of videos with the infrared camera was started when the temperature inside the freezer dropped below 0 °C. In the experiments with *J. nudiflorum* and the detached inflorescences of *G. nivalis* and *Muscari* sp. a macro lens (LW 64/150), which achieves a resolution of 200 µm, was used. The infrared camera was connected via a FireWire connection to a computer that recorded the data. The infrared camera was operated and the data analyzed using the ThermaCAM^TM^ Researcher software (FLIR Systems AB, Danderyd, Sweden).

If necessary for the evaluation, a differential infrared thermal analysis (infrared differential thermal analysis—IDTA) was carried out with the data or data sequences. The IDTA makes it possible to make even very small temperature differences visible by comparing the current temperature with the temperature before the freezing event. This is done by subtracting the temperatures of a data sequence from the temperature of a reference image. As a result, very small temperature differences can be displayed and disruptive background noise can be suppressed [9].

### 5.4. Ice Tolerance Test

Freezing treatment was conducted as described in Section 5.2, except for the cooling rate, which was set to 2 K·h^−1^. The freezer was equipped with a special lid made of Plexiglas that allowed inspection of samples throughout the whole freezing treatment. The lid had additionally two holes where thermally insulated gloves were inserted. By this, samples could be manipulated during freezing inside of the cooling compartment without any temperature changes. By inspection through the Plexiglas lid, it was not always clearly visible if samples were already frozen, as often no clear change in the appearance of samples occurred by ice nucleation. The question whether samples were already ice nucleated or still supercooling could be checked by touching the samples with a preparation needle. The touching test revealed unambiguously if a sample was frozen or still supercooled. On tablets (30 × 20 cm), 20 inflorescences of each species were laid out on wet paper towels and altogether were covered with cling film. Then the samples were subjected to the freezing treatment, and when the sublethal target temperature of −5 °C was reached, the samples were checked to see if they were supercooled or frozen. Thereafter, samples were thawed, kept at room temperature and moderate illumination, and checked for frost damage the day after. As samples were either completely uninjured or got killed by the treatment, viability was assessed by visual inspection and scoring either 0% or 100% damage. Flowers were termed ice tolerant when despite being stiffly frozen at −5 °C, they remained completely undamaged—or were termed ice susceptible when they survived in the supercooled state at −5 °C but were killed if flowers were iced in the test.

### 5.5. Microscopical Investigations

Samples of the species, for which structural ice barriers were identified according to IDTA, were observed with a reflected-light microscope [BXFM-F, Olympus Optical Co., Tokyo, Japan; see [40]]. Cross-sections were made in a longitudinal or horizontal direction using a hand-microtome (GLS 1, Schenkung Dapples, Switzerland) in the area where ice barriers are located. Pictures were taken with a still camera (UC90, Olympus Optical Co., Tokyo, Japan) mounted to the top of the microscope.

### 5.6. Data Analysis

Ice nucleation temperatures of flowers vs. vegetative parts obtained within one experiment were compared by linear mixed-effects models (LME) implemented in the function “lmer” in the package “lmerTest” [41]. LME allow us to consider that ice nucleation temperature of flower(s) and vegetative part(s) were nested per individual and/or result from different experimental runs (*G. nivalis*). For each experiment, ice nucleation temperatures of vegetative vs. reproductive plant parts was compared, regressing plant parts as fixed and individual as random factors on the ice nucleation temperature. For the experiment with *J. nudiflorum*, in which only one twig was used and for the second experiment with *Muscari* sp., in which only two flower stalks were used, no statistical comparison was possible. For the first experiment with *G. nivalis*, where the assignment of leaves to particular individuals was impossible (Figure 2) an ANOVA was applied. To compare ice nucleation of roots, vegetative parts, and flowers of *G. nivalis*, obtained by three experiments, a LME was applied, regressing the plant part as fixed and experimental run as a random factor on ice nucleation temperature. To test for interspecific differences in ice nucleation temperatures, data were compared by a linear model regressing the plant part of a species on ice nucleation temperature. The second experiment with *Muscari* sp. was excluded from this comparison, as another cooling rate was applied in this experiment. We checked the model assumption of normality and homogeneity of variance by diagnostic plots and homogeneity of variance by Levene´s test for all analyses. Parameters were estimated by optimizing the restricted maximum likelihood criterion in LME. All analyses were performed in R at a significance level of *p* < 0.05 [42].

## Figures and Tables

**Table 1:** Ice tolerance of reproductive and vegetative organs of the investigated species. After controlled freezing at a rate of −2 k·h^−1^ from +2 °C down to −5 °C, frost injuries were assessed in samples that either were stiffly frozen after ice nucleation or had remained supercooled (N = 20). Additionally, the existence of a structural ice barrier between reproductive and vegetative organs is indicated. Values are means and standard deviation of ice nucleation temperatures in different organs as determined in IDTA experiments.

Taxon	Ice Tolerance of		Structural Ice Barrier	Ice Nucleation Temperature of	
	Flowers	Leaves/Stems		Flowers	Leaves/Stems
*Anemone nemorosa* L.	-	+	+	−7.6 ± 1.1	−6.3 ± 1.1
*Galanthus nivalis* L.	+ ^1^	+	-	−5.2 ± 0.2	−6.1 ± 0.5 (−2.4 ± 0.8) ^4^
*Jasminum nudiflorum* Lindl.	-	+	+	−9.2 ²	−4.1 ± 1.8
*Muscari* sp. ³	-	+	+	−17.9 ± 3.0	−8.3 ± 1.8
*Scilla forbesii* (Baker) Speta	+	+	-	−10.7 ± 0.3	−9.9 ± 1.2

^1^ Except for tepals that could show frost injuries when ice nucleated. ² one shoot was used in the experiment. ³ results of the first experiment only, as another cooling rate was applied in the second experiment. ^4^ excavated roots.

**Table 2:** The investigated plant species were collected in the Botanical Garden of the Univeristy Innsbruck, or in case of *Muscari* bought as potted plants in a garden center. The species differ in their growth form [37], life form [38], vertical distribution [37] (except for *J. nudiflorum* [39]), and flowering time [37].

Taxon	Growth Form	Life Form	Vertical Distribution	Flowering Time
*Anemone nemorosa* L.	Perennial herb	Rhizome geophyte	colline-montane (subalpine) (−2000 m)	March–April/May
*Galanthus nivalis* L.	Bulb-forming perennial herb	Geophyte	colline-montane (100–1400 m)	January–March (April)
*Jasminum nudiflorum* Lindl.	Perennial woody shrub	Deciduous shrub	colline-subalpine (800–4500 m)	December–April
*Muscari* sp.	Bulb-forming perennial herb	Geophyte	colline-montane (subalpine) (−1200 m)	April–May
*Scilla forbesii* (Baker) Speta	Bulb-forming perennial herb	Geophyte	colline-montane	March–April

## Data Availability

Data is available on request from the correspondence author.

## Supplementary Materials

The following are available online at https://www.mdpi.com/article/10.3390/plants10051031/s1, Figure S1: Ice nucleation temperatures, Table S1: Ice nucleation temperatures (Statistical results). Supplementary Figure S1. Ice nucleation temperatures determined in flowers (“F”, white boxes), leaves (“L”, light-grey), roots (“R”, dark-grey), stems (“S”), and/or inflorescences together with stalks (“FS”, white) of *A. nemorosa* L. (“AN”), *G. nivalis* L. (“GN”), *J. nudiflorum* Lindl. (“JN”), *Muscari* sp. (“MA”), and *Scilla forbesii* (Baker) Speta (“SF”). Asterisks indicate significant differences (*p* < 0.05) from the intercept in a linear model, where the plant part of a species was regressed on ice nucleation temperatures. Box plots show medians and the 25th and 75th percentiles. Dots outside the 1.5 × interquartile ranges represent outliers. Supplementary Table S1. Coefficients of a linear model relating the plant part of a species to ice nucleation temperature. Flowers of *A. nemorosa* were used as baseline levels in the model.

## References

[1] Sakai A., Larcher W. (1987). Frost Survival of Plants. Responses and Adaptation to Freezing Stress.

[2] Neuner G., Erler A., Ladinig U., Hacker J., Wagner J. (2013). Frost resistance of reproductive tissues during various stages of development in high mountain plants. Physiol. Plant..

[3] Ladinig U., Hacker J., Neuner G., Wagner J. (2013). How endangered is sexual reproduction of high-mountain plants by summer frosts? Frost resistance, frequency of frost events and risk assessment. Oecologia.

[4] Neuner G. (2014). Frost resistance in alpine woody plants. Front. Plant Sci..

[5] Menzel A. (2000). Trends in phenological phases in europe between 1951 and 1996. Int. J. Biometeorol..

[6] Cara Donna P.J., Bains J.A. (2016). Frost sensitivity of leaves and flowers of subalpine plants is related to tissue type and phenology. J. Ecol..

[7] Hamann E., Denney D., Day S., Lombardi E., Jameel M.I., MacTavish R., Anderson J.T. (2021). Review: Plant eco-evolutionary responses to climate change: Emerging directions. Plant Sci..

[8] Stegner M., Lackner B., Schäfernolte T., Buchner O., Xiao N., Gierlinger N., Holzinger A., Neuner G. (2020). Winter nights during summer time: Stress physiological response to ice and facilitation of freezing cytorrhysis by elastic cell wall components in leaves of a nival species. Int. J. Mol. Sci..

[9] Hacker J., Neuner G. (2007). Ice propagation in plants visualized at the tissue level by idta (infrared differential thermal analysis). Tree Physiol..

[10] Hacker J., Neuner G. (2008). Ice propagation in dehardened alpine plant species studied by infrared differential thermal analysis (idta). Arct. Antarct. Alp. Res..

[11] Carter J., Brennan R., Wisniewski M. (2001). Patterns of ice formation and movement in blackcurrant. HortScience.

[12] Workmaster B.A.A., Palta J.P., Wisniewski M. (1999). Ice nucleation and propagation in cranberry uprights and fruit using infrared video thermography. J. Am. Soc. Hortic. Sci..

[13] Kuprian E., Briceno V., Wagner J., Neuner G. (2014). Ice barriers promote supercooling and prevent frost injury in reproductive buds, flowers and fruits of alpine dwarf shrubs throughout the summer. Environ. Exp. Bot..

[14] Kuprian E., Tuong T., Pfaller K., Wagner J., Livingston D., Neuner G. (2016). Persistent supercooling of reproductive shoots is enabled by structural ice barriers being active despite an intact xylem connection. PLoS ONE.

[15] Hacker J., Ladinig U., Wagner J., Neuner G. (2011). Inflorescences of alpine cushion plants freeze autonomously and may survive subzero temperatures by supercooling. Plant Sci..

[16] Quamme H.A. (1978). Mechanism of supercooling in overwintering peach flower buds. J. Am. Soc. Hortic. Sci..

[17] Ishikawa M., Sakai A. (1981). Freezing avoidance mechanisms by supercooling in some rhododendron flower buds with reference to water relations. Plant Cell Physiol..

[18] Ashworth E., Davis G. (1984). Ice nucleation within peach trees. J. Am. Soc. Hortic. Sci..

[19] Chalker-Scott L. (1992). Disruption of an ice-nucleation barrier in cold hardy *azalea* buds ny sublethal heat stress. Ann. Bot..

[20] Wisniewski M., Davies G. (1989). Evidence for the involvment of a specific cell wall layer in regulation of deep supercooling of xylem parenchyma. Plant Physiol..

[21] Quamme H.A., Su W.A., Veto L.J. (1995). Anatomical features facilitating supercooling of the flower within the dormant peach flower bud. J. Am. Soc. Hortic. Sci..

[22] Jones K., McKersie D., Paroschy J. (2000). Prevention of ice propagation by permeability barriers in bud axes of *vitis vinifera*. Can. J. Bot..

[23] Neuner G., Hacker J., Lütz C. (2012). Ice Formation and Propagation in Alpine Plants. Plants in Alpine Regions: Cell Physiology of Adaptation and Survival Strategies.

[24] Endoh K., Kasuga J., Arakawa K., Ito T., Fujikawa S. (2009). Cryo-scanning electron microscopic study on freezing behaviors of tissue cells in dormant buds of larch (larix kaempferi). Cryobiology.

[25] Ashworth E.N. (1984). Xylem development in prunus flower buds and the relationship to deep supercooling. Plant Physiol..

[26] Ashworth E.N., Willard T.J., Malone S.R. (1992). The relationship between vascular differentiation and the distribution of ice within forsythia flower buds. Plant Cell Environ..

[27] Julian C., Herrero M., Rodrigo J.F. (2007). Flower bud drop and pre-blossom frost damage in apricot (*Prunus armeniaca* l.). J. Appl. Bot. Food Qual..

[28] Pramsohler M., Neuner G. (2013). Dehydration and osmotic adjustment in apple stem tissue during winter as it relates to the frost resistance of buds. Tree Physiol..

[29] Kasuga J., Hashidoko Y., Nishioka A., Yoshiba M., Arakawa K., Fujikawa S. (2008). Deep supercooling xylem parenchyma cells of katsura tree (cercidiphyllum japonicum) contain flavonol glycosides exhibiting high anti-ice nucleation activity. Plant Cell Environ..

[30] Ishikawa M., Ishikawa M., Toyomasu T., Aoki T., Price W.S. (2015). Ice nucleation activity in various tissues of rhododendron flower buds: Their relevance to extraorgan freezing. Front. Plant Sci..

[31] Körner C. (2003). Alpine Plant Life. Functional Plant Ecology of High Mountain Ecosystems.

[32] Fernández-Marín B., Arzac M.I., López-Pozo M., José Manuel L., Roach T., Stegner M., Neuner G., García-Plazaola J. (2021). Frozen in the dark: Interplay of night-time activity of xanthophyll cycle, xylem attributes, and desiccation tolerance in fern resistance to winter. J. Exp. Bot..

[33] Marcante S., Sierra-Almeida A., Spindelbock J., Erschbamer B., Neuner G. (2012). Frost as a limiting factor for recruitment and establishment of early development stages in an alpine glacier foreland?. J. Veg. Sci..

[34] Till O. (1956). Über die frosthärte von pflanzen sommergrüner laubwälder. Flora.

[35] Taschler D., Neuner G. (2004). Summer frost resistance and freezing patterns measured in situ in leaves of major alpine plant growth forms in relation to their upper distribution boundary. Plant Cell Environ..

[36] Neuner G., Huber B., Plangger A., Pohlin J.M., Walde J. (2020). Low temperatures at higher elevations require plants to exhibit increased freezing resistance throughout the summer months. Environ. Exp. Bot..

[37] Lauber K., Wagner G. (2007). Flora Helvetica.

[38] Raunkiaer C. (1934). The Life Forms of Plants and Statistical Plant Geography.

[39] Flora of China. http://www.efloras.org/florataxon.aspx?flora_id=2&taxon_id=200017786.

[40] Stegner M., Wagner J., Neuner G. (2020). Ice accommodation in plant tissues pinpointed by cryo-microscopy in reflected-polarised-light. Plant Methods.

[41] Kuznetsova A., Brockhoff P.B., Christensen R.H.B. (2017). Lmertest package: Tests in linear mixed effects models. J. Stat. Softw..

[42] R Core Team (2019). R: A Language and Environment for Statistical Computing.

