# Supplementary material for: Protective Role of Ice Barriers: How Reproductive Organs of Early Flowering and Mountain Plants Escape Frost Injuries
Source: Plants (Basel). 2021 May 20;10(5):1031. doi: 10.3390/plants10051031 (PMC8161042; doi:10.3390/plants10051031)
Supplement: Supplementary file 1 [file plants-10-01031-s001.zip › plants-1222573-supplementary.pdf]

Supplementary Materials: The following are available online at www.mdpi.com/xxx/s1, Figure S1: Ice nucleation temperatures, Table S1: Ice nucleation temperatures (Statistical results).

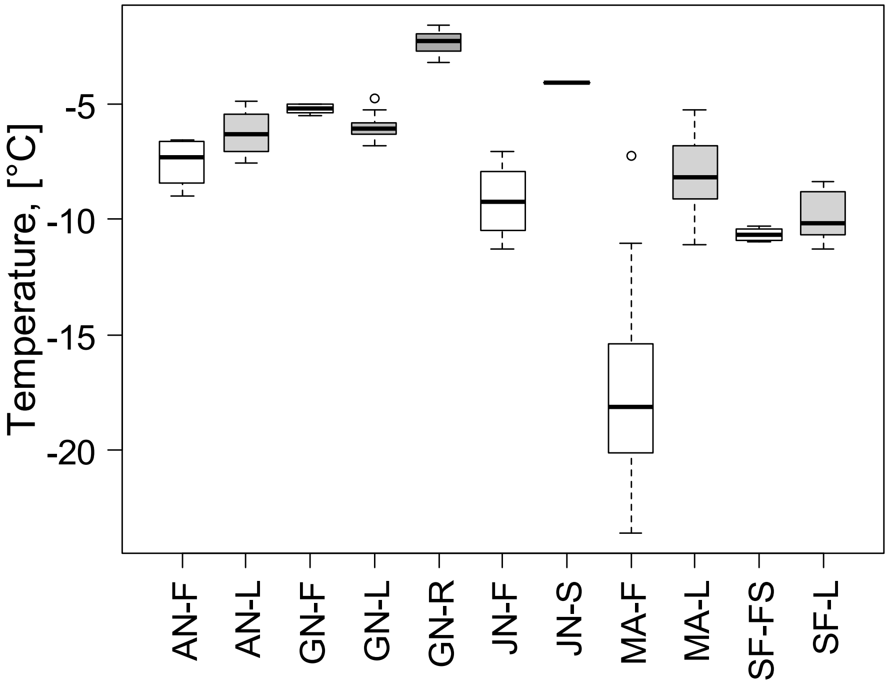

Supplementary Figure S1. Ice nucleation temperatures determined in flowers ("F", white boxes), leaves ("L", light-grey), roots ("R", dark-grey), stems ("S"), and/or inflorescences together with stalks ("FS", white) of *Anemone nemorosa* L. ("AN"), *Galanthus nivalis* L. ("GN"), *Jasminum nudiflorum* Lindl. ("JN"), *Muscari sp.* ("MA"), and *Scilla forbesii* (Baker) Speta ("SF"). Asteriks indicate significant differences ($p < 0.05$) from the intercept in linear model, where the plant part of a species was regressed on ice nucleation temperatures. Box plots show medians and the 25th and 75th percentiles. Dots outside the 1.5 × interquartile ranges represent outliers.

Supplementary Table S1. Coefficients of a linear model relating the plant part of a species to ice nucleation temperature. Flowers of *A. nemorosa* were used as baseline levels in the model.

Plant species and Part	Coefficient ± SE	t value	p-value
Anemone nemorosa L. flowers	-7.6 ± 1.3	-5.627	**<0.001**
Anemone nemorosa L. leaves	-6.3 ± 1.9	0.672	0.502
Galanthus nivalis L. flowers	-5.2 ± 1.6	1.482	0.139
Galanthus nivalis L. leaves	-6.1 ± 1.5	1.010	0.313
Galanthus nivalis L. roots	-2.4 ± 2.1	2.529	**0.012**
Jasminum nudiflorum Lindl. flowers	-9.2 ± 1.9	-0.883	0.378
Jasminum nudiflorum Lindl.shoot	-4.1 ± 3.0	1.150	0.251
Muscari sp. flowers	-17.9 ± 1.4	-7.637	**<0.001**
Muscari sp. leaves	-8.3 ± 1.6	-0.472	0.637
Scilla forbesii (Baker) Speta flowers	-10.7 ± 1.9	-1.647	0.101
Scilla forbesii (Baker) Speta leaves	-9.9 ± 1.8	-1.294	0.197
